# Urinary levels of organophosphate flame retardants metabolites in a young population from Southern Taiwan and potential health effects

**DOI:** 10.3389/fendo.2023.1173449

**Published:** 2023-06-02

**Authors:** Feng-Shun Chen, Chih-Cheng Chen, Ching-Chang Tsai, Jian-He Lu, Huey-Ling You, Ching-Mei Chen, Wan-Ting Huang, Kai-Fan Tsai, Fu-Jen Cheng, Chia-Te Kung, Shau-Hsuan Li, Chin-Chou Wang, Yu-Che Ou, Wen-Chin Lee, Yu-Ting Chang, Fahimah Hashim, How-Ran Chao, Liang-Jen Wang

**Affiliations:** ^1^ Section of Neonatology, Department of Pediatrics, Kaohsiung Chang-Gung Memorial Hospital, Kaohsiung, Taiwan; ^2^ Department of Early Childhood Care and Education, Cheng-Shiu University, Kaohsiung, Taiwan; ^3^ Department of Obstetrics and Gynecology, Kaohsiung Chang Gung Memorial Hospital and Chang Gung University College of Medicine, Kaohsiung, Taiwan; ^4^ Department of Environmental Science and Engineering, College of Engineering, National Pingtung University of Science and Technology, Neipu, Taiwan; ^5^ Department of Laboratory Medicine, Kaohsiung Chang Gung Memorial Hospital and Chang Gung University College of Medicine, Kaohsiung, Taiwan; ^6^ Division of Nephrology, Department of Internal Medicine, Kaohsiung Chang Gung Memorial Hospital and Chang Gung University College of Medicine, Kaohsiung, Taiwan; ^7^ Department of Emergency Medicine, Kaohsiung Chang Gung Memorial Hospital and Chang Gung University College of Medicine, Kaohsiung, Taiwan; ^8^ Division of Hematology-Oncology, Department of Internal Medicine, Kaohsiung Chang Gung Memorial Hospital and Chang Gung University College of Medicine, Kaohsiung, Taiwan; ^9^ Department of Occupational Medicine, Kaohsiung Chang Gung Memorial Hospital and Chang Gung University College of Medicine, Kaohsiung, Taiwan; ^10^ Universiti Malaysia Terengganu, Kuala Terengganu, Malaysia; ^11^ Institute of Food Safety Management, College of Agriculture, National Pingtung University of Science and Technology, Pingtung, Taiwan; ^12^ Department of Child and Adolescent Psychiatry, Kaohsiung Chang Gung Memorial Hospital and Chang Gung University College of Medicine, Kaohsiung, Taiwan

**Keywords:** organophosphate flame retardants (OPFRs), OPFR metabolites, urine, a broad-spectrum pediatric population, newborn, hazardous chemicals

## Abstract

**Background:**

Organophosphate flame retardants (OPFRs) are widely distributed in the environment and their metabolites are observed in urine, but little is known regarding OPFRs in a broad-spectrum young population from newborns to those aged 18 years.

**Objectives:**

Investigate urinary levels of OPFRs and OPFR metabolites in Taiwanese infants, young children, schoolchildren, and adolescents within the general population.

**Methods:**

Different age groups of subjects (n=136) were recruited from southern Taiwan to detect 10 OPFR metabolites in urine samples. Associations between urinary OPFRs and their corresponding metabolites and potential health status were also examined.

**Results:**

The mean level of urinary Σ_10_ OPFR in this broad-spectrum young population is 2.25 μg/L (standard deviation (SD) of 1.91 μg/L). Σ_10_ OPFR metabolites in urine are 3.25 ± 2.84, 3.06 ± 2.21, 1.75 ± 1.10, and 2.32 ± 2.29 μg/L in the age groups comprising of newborns, 1-5 year-olds, 6-10 year-olds, and 11-18 year-olds, respectively, and borderline significant differences were found in the different age groups (*p*=0.125). The OPFR metabolites of TCEP, BCEP, DPHP, TBEP, DBEP, and BDCPP predominate in urine and comprise more than 90% of the total. TBEP was highly correlated with DBEP in this population (r=0.845, *p*<0.001). The estimated daily intake (EDI) of Σ_5_OPFRs (TDCPP, TCEP, TBEP, TNBP, and TPHP) was 2,230, 461, 130, and 184 ng/kg bw/day for newborns, 1-5 yr children, 6-10 yr children, and 11-17 yr adolescents, respectively. The EDI of Σ_5_OPFRs for newborns was 4.83-17.2 times higher than the other age groups. Urinary OPFR metabolites are significantly correlated with birth length and chest circumference in newborns.

**Conclusion:**

To our knowledge, this is the first investigation of urinary OPFR metabolite levels in a broad-spectrum young population. There tended to be higher exposure rates in both newborns and pre-schoolers, though little is known about their exposure levels or factors leading to exposure in the young population. Further studies should clarify the exposure levels and factor relationships.

## 1 Introduction

Organophosphate flame retardants (OPFRs), which are a group of semi-volatile organic compounds, are a class of endocrine-disrupting chemicals (EDCs). OPFRs have gained increasing attention due to their high usage following the phaseout and strict regulations on brominated flame retardants (BFRs), such as polybrominated diphenyl ethers (PBDEs). Following the banning or voluntary phaseout of BFRs in the European and American markets, OPFRs are now the primary flame retardant (FR) products or alternatives to BFRs (1, 2). OPFRs are thermally stable organic compounds with organic esters of phosphoric acid, including alkyl-chain, aryl-group, halogenated, or non-halogenated OPFRs (1). Since OPFRs are used in many housing materials and consumer products, their presence in indoor dust or air has been widely studied. Linked to the ubiquitous presence of these compounds in indoor environments and their potential for adverse health effects, there is increasing concern about human exposure to OPFRs, particularly for infants, toddlers, and young children. One of the major human exposure pathways for OPFRs is house dust ingestion, especially for infants, toddlers, and young children, who are at home for longer periods of time. Compared with adults, the young population is more sensitive to OPFRs.

The observed adverse effects of human exposure to OPFRs include disruption of normal endocrine and reproductive functions, neurodevelopment, and cancer (1, 3–5). Compared with BFRs, most scientists recognize that OPFRs have fewer adverse health effects, including cardiovascular, neurological, and reproductive toxicity, disruption of endocrine hormones, and carcinogenicity (6). OPFRs induce damaging changes in immunity, metabolism, genetics, and endocrine activity (1, 7). Prolonged exposure and the accumulation of organophosphate esters in the human body may elicit various adverse effects, including kidney toxicity, neurotoxicity, reproductive toxicity, carcinogenicity, and endocrine disruption (8–10). Chlorinated OPFRs, such as tris(1,3-dichloro-2-propyl) phosphate (TDCPP), tris(2-chloro-1-methylethyl) phosphate (TCPP), and tris(2-chloroethyl) phosphate (TCEP) have been shown to be neurotoxic and carcinogenic (11, 12). TDCIPP can easily enter the bloodstream, liver, kidneys, and testicles and can induce tumors (11). TDCIPP levels in house dust were associated with lower levels of male thyroid hormone and elevated levels of prolactin (13). In women with higher levels of urinary OPFR metabolites, there were significant decreases in fertilization and implantation rates, successful pregnancy, and live birth rates (14).

OPFRs are readily metabolized, and studies have found ubiquitous detection of OPFR metabolites in child and adult urine, suggesting that these metabolites are convenient biomarkers of OPFR exposure (15, 16). Urine as a non-invasive specimen may thus be a good indicator for estimating the human burden of OPFRs, especially since cord blood, blood, or breast milk are invasive and more difficult to obtain than urine. The half-lives of OPFRs and their metabolites were estimated in animal models to be relatively short (hours to days) due to the easy conversion of OPFRs to OPFR metabolites (3, 17–19). OPFR metabolites, including mono- or diesters, are primarily excreted in urine and other metabolic products via hydroxylation and conjugation were possibly excreted by the excretion pathway such as feces or expired air (7, 19). Most studies detect urinary OPFRs and OPFR metabolites because urinary OPFRs and OPFR metabolites are frequently used in biomonitoring surveys from various countries, including China, the US, Germany, Norway, Australia, Taiwan, and Japan (20–34).

Currently, there are few reports on OPFRs in indoor dust or PM_2.5_ and OPFR metabolites in children’s urine in Taiwan. This study seeks to survey urinary levels of OPFRs and OPFR metabolites in the young population from newborns to adolescents under 18 years old. The distribution of OPFRs and OPFR metabolites and their correlations between OPFRs and OPFR metabolites are also examined. Health risks are evaluated for the different age groups.

## 2 Materials and methods

This report is part of a cohort study examining the adverse health effects for the young population after exposure to OPFRs. It establishes background levels of urinary OPFRs and OPFR metabolites for a broad-spectrum young population including newborns, toddlers, young children, school children, and adolescents under 18 years old in southern Taiwan. The studied subjects participated in a multidisciplinary pediatric care program between November 2020 and April 2022, with participants recruited from the pediatric outpatient clinics of Kaohsiung Chang Gung Memorial Hospital (KCGMH). This cohort study was approved by the Institutional Review Boards (IRB) of the KCGMH Human Ethical Committee and all ethical standards were followed as outlined in the Helsinki Declarations (IRB number: 202001028A3). All subjects, subjects’ parents, or subjects’ guardians read a detailed explanation of the possible consequences and then signed informed consent forms before the enrolment. The subjects were selected based on the following criteria: the children were age-matched to this study group and had no diagnosis of neurological or physiological disease.

The analytical methods to determine OPFRs and their metabolites in urine are described in a previous study (35). First-void morning urine samples were gathered in polypropylene plastic cups or urine collection bags (Deltalab, Barcelona, Spain) and after enrollment, the samples were transferred to 1.5 mL amber microcentrifuge tubes (ExtraGene, Taichung, Taiwan) stored at -80°C before chemical analysis. The 200-μL urine samples were mixed with 20 μL β-glucuronidase enzyme (Sigma-Aldrich, USA) at a PH value of 6.5 and spiked internal standards of isotope-labeled TDCPP and DNBP (Sigma-Aldrich, USA). The urinary solution was passed by solid-phase extraction (SPE) cartridge after a 2-hour incubation at 37 °C, and the SPE cartridge was conditioned with methanol (1 mL) and ultrapure water (1 mL). A mixture of 0.5% formic acid and 95% acetonitrile in 250 μL was added to the extract. Five OPFRs (TDCPP, TCEP, tris(2-butoxyethyl)phosphate (TBEP), tri-*n*-butyl phosphate (TNBP), and triphenyl phosphate (TPHP)) and five OPFR metabolites (Bis(1,3-dichloro-2-propyl) phosphate (BDCPP), Bis(chloroethyl) phosphate (BCEP), Di-(2-butoxyethyl) phosphate (DBEP), DNBP (Di-n-butyl phosphate), and Diphenyl phosphate (DPHP)) in urine were determined by ultra-performance liquid chromatography-tandem mass spectrometry (UPLC-MS/MS). The elute was then analyzed by a Waters Acquity Ultra-Performance Liquid Chromatography coupled with a Waters Xevo TQ-XS mass spectrometer (Milford, USA) using the positive or negative electrospray ionization mode with the series columns of Waters Acquity UPLC BEH Phenyl column (2.1 mm × 50 mm, 1.7 μm particle size) and Waters XBridge BEH C18 Direct Connect HP isolated column (2.1 mm × 30 mm, 10 µm particle size) in KCGMH. The injection volume and flow rate of the column were 2 μL and 0.3 mL/min, respectively. The two mobile phases were solvent A (0.5% formic acid in water) and B (0.5% formic acid in methanol) and the gradient in UPLC-MS/MS was linearly decreased from 95% to 50% of solvent A within 0.75 minutes. Then, solvent A was gradient decreased from 50% to 0% in 3 minutes to maintain 100% solvent B for another 4.5 minutes. Finally, the gradient was distinctly increased to 95% solvent A. Mass spectrometry was equipped with an electrospray ionization probe interface with 3 and 2.6 kV for electrospray mode (ES) with positive (ES+) and negative (ES-) mode, respectively. Quality assurance and control (QA/QC) of this analytical method includes blanks (field and lab blanks), recovery tests, precision, limits of detection (LODs), and limits of quantification (LOQs). Recoveries of spiked internal labeled standards, surrogate, blank, and precision and recovery of the relevant standards were between 90.7 and 11.964% within the acceptable criteria (70-130%). LODs and LOQs were defined as the ratios of signal to noise (S/N) higher than 3 and 10, respectively. LOQs for TDCPP, TCEP, TBEP, TnBP, TPHP, BDCPP, BBOEP, DnBP, and DPHP were 0.02 μg/L, and LOQ of BCEP was 0.05 μg/L.

Estimated daily intake (EDI) of TDCPP, TCEP, TBEP, TNBP, and TPHP from BDCPP, BCEP, DBEP, DNBP, and DPHP excretion, the molar fraction is calculated by a fraction of OPFRs converted to OPFR metabolites and excreted in urine per day (36). EDIs of our subjects are estimated by the following equation with minor modification (26, 27, 33, 34):


$$\text{EDI}{\text{s}}_{\text{opfrs}}(\text{ng}/\text{kg bw}/\text{day})=\text{urinary }{\text{C}}_{\text{opfr metabolite }}\text{x U}{\text{V}}_{\text{excr}})/{\text{F}}_{\text{ue }}\text{x children weight})\;\text{x}\;\left(\text{MW of OPFRs}/\text{MW of the corresponding OFPR metabolite}\right)$$

where urinary C_opfr metabolite_ (μg/L) is the mean value of an individual OPFR metabolite; UV_excre_ is defined as the daily excretion volume of urine (newborn: 0.3 L, 1-5 yr: 0.45 L, 6-10 yr: 0.7 L, and 11-18 yr: 1.2 L) (37); F_ue_ is recognized as the molar fraction of OPFR metabolites in urinary excretion with the corresponding parent OPFRs (26, 27, 34); MWs are molecular weights of TDCPP, TCEP, TBEP, TNBP, and TPHP or their corresponding metabolites of BDCPP, BCEP, DBEP, DNBP, and DPHP. Children’s weights (kg) were 3.32, 15.2, 30.0, and 55.9 kg for newborns, 1-5 yr children, 6-10 yr school children, and 11-17 adolescents, respectively, according to statistics from the Taiwan Ministry of Health and Welfare.

Measurements of OPFRs and OPFR metabolites lower than LODs were set as zero for statistical analysis. The descriptive analysis determined the mean and standard deviations (SD) of urinary OPFRs and OPFR metabolites. Urinary OPFRs and OPFR metabolites did not fulfill the normal distribution to examine the bivariate correlations among OPFR and OPFR metabolite species by Spearman’s rho correlation coefficients tests. Mann-Whitney *U* and Kruskill-Wallis *H* non-parametric methods determined differences among age groups. All statistical analyses were performed using Statistical Product and Service Solutions, version 12.0.

## 3 Results

This study is a urinary OPFRs survey research to measure urinary levels of OPFRs (TDCPP, TCEP, TBEP, TNBP, and TPHP) and OPFR metabolites (BDCPP, BCEP, DBEP, DNBP, and DPHP) in a broad-spectrum young population from southern Taiwan. The descriptive analysis of sex, height, weight, and BMI or Quetelet’s index is shown in **Table 1**. **Table 1** also presents a descriptive analysis of the OPFRs and their metabolites in a sample of 135 participants from four different broad-spectrum young populations. The mean Σ_10_OPFR level for the sample was 2.25 μg/L, with a standard deviation (SD) of 1.91 μg/L, indicating that the data was widely dispersed as SD. The sample was diverse in terms of sex, with 71 male and 65 female participants. Newborns (3.25 ± 2.84 μg/L) did not have a significantly higher Σ_10_ OPFRs level in urine than young children aged 1-5 years (yrs) (3.06 ± 2.21), school children aged 6-11 yrs (1.75 ± 1.10 μg/L), and adolescents aged 12-17 yrs (2.32 ± 2.29 μg/L). Results showed that the highest levels of OPFRs were found in the newborns for the compounds TCEP, BCEP, TBEP, DBEP, and DPHP, with mean ± SD of 1.13 ± 1.51, 0.705 ± 1.00, 0.424 ± 0.933, 0.406 ± 1.32, and 0.395 ± 0.592 μg/L, respectively. Similarly, young children in the 1-5 yrs category may also be particularly vulnerable to OPFR exposure, with the highest levels in BCEP (0.680 ± 0.834), TBEP (0.634 ± 0.866), DPHP (0.628 ± 0.871), and DBEP (0.469 ± 0.752). Among the OPFRs measured in the 1-5 yrs children, the compounds DPHP, TBEP, BCEP, and TCEP had the highest levels of OPFRs, with mean values ranging from 0.290 to 0.461 μg/L (SD from 0.380 to 0.773 μg/L). Finally, for the 11-18 yrs adolescents, the compounds DPHP, BCEP, and BDCPP had the highest levels of OPFRs, with a mean between 0.514 to 0.585 μg/L (SD: 0.551-1.20 μg/L). TBEP and its metabolite, DBEP, showed significant and margin-significant differences between newborns (TBEP: 0.424 ± 0.933 μg/L; DBEP: 3.25 ± 2.84 μg/L) and adolescents (TBEP: 0.217 ± 0.292 μg/L, *p*=0.026; DBEP: 0.149 ± 0.214 μg/L, *p*=0.054), respectively. The study found that the group’s average height was 86.3 cm with a range of ± 43.2 cm, the average weight was 15.9 kg with a range of ± 17.7 kg, and the average BMI was 14.5 with a range of ± 3.56.

**Table 1:** Descriptive analysis for the young population in the present study (n=135).

	Total (n = 135)	Newborn (n=66)	1 - 5 years (n=19)	6 - 10 years (n=30)	11 - 18 years (n=20)	p-value
Age (mean ± SD)	4.33 ± 5.05	0.141 ± 0.0580	3.59 ± 1.14	8.16 ± 1.33	13.4 ± 2.00	
Sex, n (%)						
Male	71 (52.2)	34 (51.5)	10 (52.6)	13 (43.3)	14 (70.0)	
Female	65 (47.8)	32 (48.5)	9 (47.4)	17 (56.7)	6 (30.0)	
Height (mean ± SD, cm)	86.3 ± 43.2	49.3 ± 2.45	98.0 ± 8.86	126 ± 9.81	158 ± 16.5	
Weight (mean ± SD, kg)	15.9 ± 17.7	2.94 ± 0.464	15.1 ± 2.90	26.0 ± 6.23	50.6 ± 12.4	
BMI or Quetelet’s index (mean ± SD)	14.5 ± 3.56	12.0 ± 1.17	15.6 ± 1.65	16.2 ± 2.57	20.0 ± 3.75	
Contaminants (ug/L) (mean ± SD)						
OPFRs and the metabolites						
BDCPP	0.177 ± 0.629	0.0530 ± 0.263	0.279 ± 0.600	0.165 ± 0.456	0.514 ± 1.31	0.789
BCEP	0.578 ± 0.824	0.705 ± 1.00	0.680 ± 0.834	0.293 ± 0.380	0.517 ± 0.551	0.154
DBEP	0.327 ± 0.980	0.406 ± 1.32	0.469 ± 0.752	0.182 ± 0.318	0.149 ± 0.214	0.054
DNBP	0.0203 ± 0.111	0.0400 ± 0.157	0.00158 ± 0.00688	0.00333 ± 0.00884	< LOD	0.201
DPHP	0.467 ± 0.725	0.395 ± 0.592	0.628 ± 0.871	0.461 ± 0.444	0.585 ± 1.20	0.454
TDCPP	0.0214 ± 0.0632	0.00900 ± 0.0320	0.0421 ± 0.0879	0.0193 ± 0.0592	0.0450 ± 0.103	0.229
TCEP	0.682 ± 1.26	1.13 ± 1.51	0.265 ± 0.796	0.290 ± 0.773	0.223 ± 0.711	0.949
TBEP	0.394 ± 0.776	0.424 ± 0.933	0.634 ± 0.866	0.295 ± 0.503	0.217 ± 0.292	0.026
TNBP	0.0380 ± 0.0505	0.0430 ± 0.0600	0.0416 ± 0.0379	0.0270 ± 0.0392	0.0330 ± 0.0383	0.171
TPHP	0.0342 ± 0.110	0.0470 ± 0.150	0.0142 ± 0.0255	0.0157 ± 0.0305	0.0380 ± 0.0698	0.490
ΣOPFRs	2.25 ± 1.91	3.25 ± 2.84	3.06 ± 2.21	1.75 ± 1.10	2.32 ± 2.29	0.125

**Figure 1:**
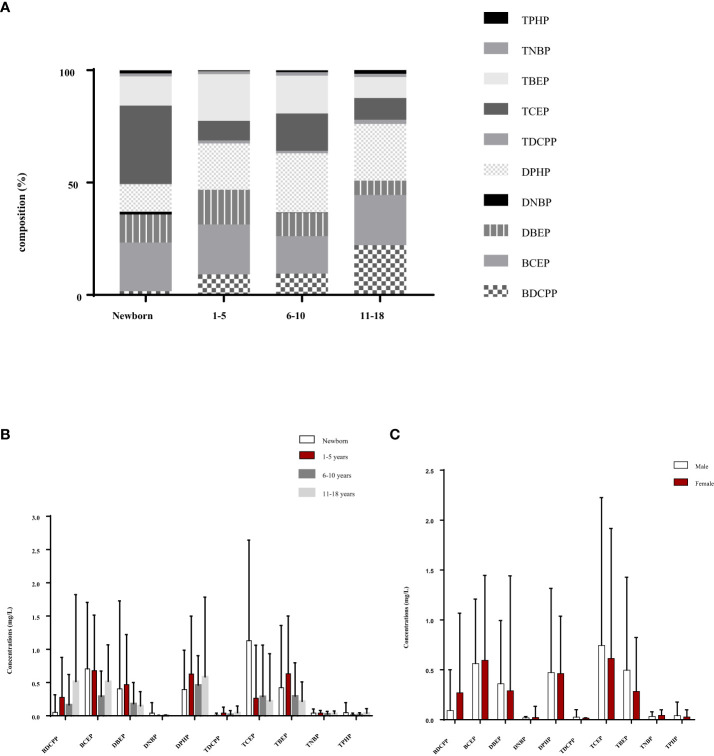
Urinary levels of OPFRs and OPFR metabolites for the young population in southern Taiwan are shown as **(A)** the percentage of OPFRs and OPFR metabolites in the young population’s urine, **(B)** distribution of urinary OPFR and OPFR metabolite levels in the different age groups and **(C)** urinary levels of OPFRs and OPFR metabolites for male and female subjects in the young population.

**Figures 1A, B** illustrate the percentage of OPFRs and OPFR metabolites found in children’s urine samples in Southern Taiwan. Overall, there were similar patterns for the age groups 1-5, 6-10, and 11-18 years, except for the newborns, which differed significantly. Studies indicated that the percentage of BDCPP in newborns was the lowest at only 1.64%, in contrast to 9.13% for young children aged 1-5 yrs, 9.42% for school children aged 6-10 yrs, and 22.2% for adolescents aged 11-18 yrs. Similarly, the percentage of DPHP was also the lowest in newborns at 12.1%, while the other categories ranged from 20.6 to 26.3%. However, for TCEP, newborns had the highest percentage at 34.7%, compared with the other groups, which were below 20% of the composition. **Figure 1C** shows the urinary levels of OPFRs and OPFR metabolites between male and female subjects. The concentrations of certain compositions, such as DBEP, DPHP, TDCPP, TCEP, TBEP, and TPHP, tend to be found more often in male subjects than female subjects. The graph shows that BCEP, TCEP, and TBEP had levels over 0.5 μg/L; whereas concentrations of DNBP, TDCPP, TNBP, and TPHP were lower, all being under 0.1 μg/L.

**Table 2** shows Spearman’s rho correlation coefficient (r) between urinary OPFRs and OPFR metabolites in the newborn group of 0.816 (*p* < 0.001) between TBEP and DBEP, indicating a strong positive relationship between the two variables. There was also a strong positive correlation between DNBP and TNBP (r= 0.509, *p <*0.001), as well as between TDCPP and TPHP (r = 0.458, *p <*0.001) and between DBEP and DPHP (r=0.384, *p*=0.001). TNBP and TPHP showed a strong positive correlation (r=0.244, p=0.048), while TCEP and TPHP exhibited a strong negative correlation (r=-0.279, *p*=0.0230). Similarly, the relationship between BCEP and DNBP was positively strong (r=0.305, *p*=0.013), whereas the relationship between BCEP and TBEP was negatively strong (r=-0.365, *p*=0.003).

**Table 2:** Spearman’s rho correlation coefficients between urinary OPFRs and OPFR metabolites in the newborn group (n=66).

Contaminants	BDCPP	BCEP	DBEP	DNBP	DPHP	TDCPP	TCEP	TBEP	TNBP
BCEP	0.0520^a^ (0.677)^b^								
DBEP	0.0330 (0.794 )	-0.108 (0.386)							
DNBP	0.0370 (0.766)	0.305* (0.013)	0.119 (0.339)						
DPHP	-0.0130 (0.915)	0.0410 (0.746)	0.384** (0.001)	0.069 (0.583)					
TDCPP	-0.132 (0.292)	-0.154 (0.217)	-0.00400 (0.976)	-0.0310 (0.803)	0.0330 (0.795)				
TCEP	-0.178 (0.152)	0.200 (0.107)	-0.0400 (0.749)	0.0150 (0.907)	-0.122 (0.328)	-0.157 (0.209)			
TBEP	-0.0500 (0.692)	-0.365** (0.003)	0.816*** (<0.001)	-0.0620 (0.620)	0.250* (0.043)	0.141 (0.259)	-0.174 (0.163)		
TNBP	-0.263 (0.0330)	0.198 (0.111)	0.150 (0.231)	0.509*** (<0.001)	0.0600 (0.631)	0.112 (0.370)	0.104 (0.406)	0.089 (0.479)	
TPHP	-0.141 (0.259)	-0.201 (0.106)	0.239 (0.054)	0.0220 (0.860)	0.0760 (0.545)	0.458*** (<0.001)	-0.279* (0.0230)	0.325** (0.008)	0.244* (0.048)

a Spearman’s rho correlation coefficient.

b (p-value).

^*^p< 0.05, ^**^p< 0.01, ^***^ p< 0.001.

**Figure 2:**
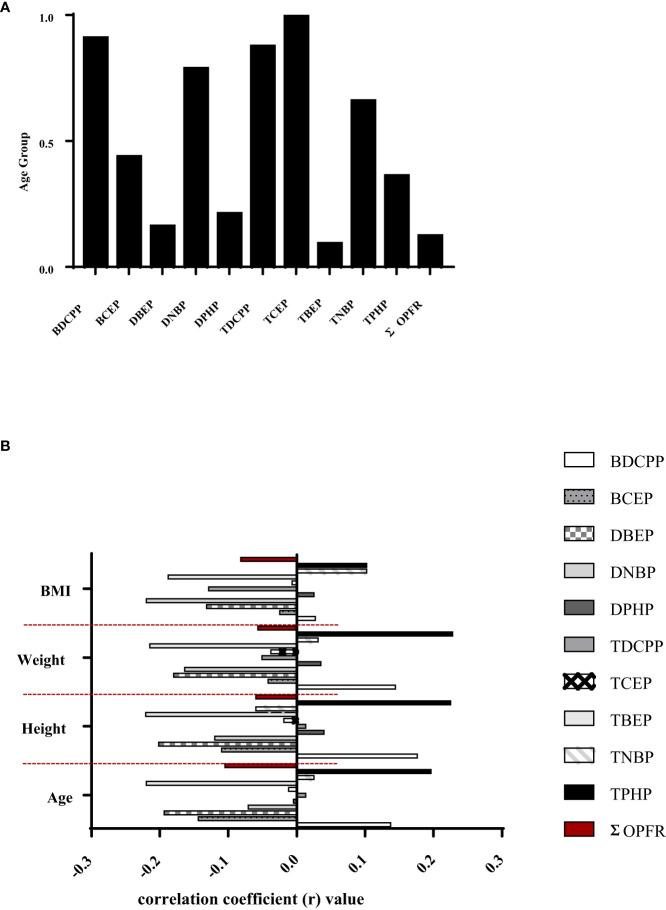
**(A)** Spearman’s rho correlation coefficients between age groups and urinary OPFRs or OPFR metabolites in the young population (n=135). **(B)** Spearman's rho correlations between urinary OPFRs or OPFR metabolites and height, weight, and BMI in youngster individuals (n=69).

**Table 3** presents the correlation coefficient between OPFR metabolites and urinary OPFRs for the 69 participants of the 1-17 yrs group. Similar to the newborn group, correlation analysis for the DBEP and TBEP relationship revealed a strong correlation, with the highest coefficient of r=0.845 and *p <*0.001. The second highest correlation was between TDCPP and TNBP, with a coefficient of r=0.374 and a *p*-value of 0.002, followed by the correlation between DBEP and TNBP (r=0.344, *p*-value=0.004). There was a significant positive relationship between BCEP and DPHP and between TBEP and TNBP, with r=0.244, *p*=0.0440, and r=0.247, *p*=0.041, respectively. In contrast, the relationships between DBEP and TCEP, as well as TCEP and TBEP, were both negative. The correlation coefficient for DBEP and TCEP was -0.260 with a *p*-value of 0.031, and for TCEP and TBEP, it was -0.296 with a *p*-value of 0.014. The age group in **Figure 2A** had a high correlation with certain OPFR metabolites in urine, such as BDCPP, DNBP, TDCPP, TCEP, and TNBP; while BCEP and TPHP were moderately related to this age group.

**Table 3:** The Spearman’s rho correlation coefficients between urinary OPFRs and OPFR metabolites in children aged 1 to 17 years (n=69).

Contaminants	BDCPP	BCEP	DBEP	DNBP	DPHP	TDCPP	TCEP	TBEP	TNBP
BCEP	-0.0850^a^ (0.487)^b^								
DBEP	-0.0700 (0.567 )	0.108 (0.375)							
DNBP	-0.121 (0.322)	-0.049 (0.692)	-0.001 (0.991)						
DPHP	0.226 (0.062)	0.244* (0.0440)	-0.009 (0.945)	0.036 (0.767)					
TDCPP	-0.196 (0.107)	-0.151 (0.216)	0.112 (0.359)	-0.0900 (0.460)	-0.0520 (0.672)				
TCEP	0.204 (0.093)	-0.020 (0.154)	-0.260* (0.031)	-0.101 (0.409)	-0.143 (0.241)	0.018 (0.886)			
TBEP	0.060 (0.626)	0.0800 (0.471)	0.845*** (<0.001)	0.0800 (0.512)	0.0530 (0.664)	0.131 (0.282)	-0.296* (0.014)		
TNBP	-0.109 (0.371)	-0.0190 (0.875)	0.344** (0.004)	-0.157 (0.197)	-0.120 (0.327)	0.374** (0.002)	0.166 (0.172)	0.247* (0.041)	
TPHP	0.200 (0.100)	0.154 (0.207)	-0.0320 (0.792)	-0.0870 (0.479)	0.209 (0.0850)	-0.211 (0.082)	-0.110 (0.366)	0.089 (0.466)	-0.0860 (0.480)

a Spearman’s rho correlation coefficient.

b (p-value).

^*^p< 0.05, ^**^p< 0.01, ^***^ p< 0.001.

**Figure 2B** depicts Spearman’s rho correlations among BMI, weight, and height for a group of young individuals. Among the OFPRs metabolites in the present study, no significant correlations with age, height, weight, and BMI are shown in **Figure 2B**. **Table 4** displays the variability of non-neonatal questionnaire responses, including seven parameters: the frequency of eating out, hand-washing habits before eating, daily consumption of beverages from plastic cups, daily consumption of microwaved food, weekly intake of seafood and large fish, and the number of daily meat servings. Among these parameters, the frequency of eating out had the strongest correlation with TCEP (*p*=0.008) and TBEP (*p*=0.042). Additionally, the hand-washing parameter showed a significant correlation with TNBP (*p*=0.049). Children who frequently ate out (≥7 times/week) had significantly higher levels of TCEP (0.643 ± 1.083 μg/L) and lower magnitudes of TBEP (0.188 ± 0.273 μg/L) in urine compared with those children who did not frequently eat out, respectively (TCEP: 0.115 ± 0.513 μg/L, *p*=0.008; TBEP: 0.473 ± 0.695 μg/L, *p*=0.042). **Table 5** presents Spearman’s rho correlation coefficients between urinary OPFRs and neonatal birth outcomes, based on a sample size of 66. The descriptive analyses are shown as the following: Quetelet’s index (12.0 ± 1.17 kg/m^2^), Apgars score 1 minute (8.97± 0.173), Apgars score 5 minutes (9.95 ± 0.210), birth length (49.3 ± 2.45 cm), birth weight (2.94 ± 0.464 kg, head circumference (49.3 ± 2.45 cm), chest circumference (31.8 ± 2.04 cm), abdominal circumference (29.4 ± 2.18 cm), and gestational age (37.9± 1.34 weeks). Significant negative relationships were observed between the urinary BDCPP chemical and birth length (r=-0.249, *p*=0.044) as well as between BDCPP and chest circumference (r=-0.261, *p*=0.034). Additionally, a negative correlation was found between DBEP and birth length, with r=-0.252 and *p*=0.041. In contrast, TCEP and birth weight exhibited a positive relationship, with r=0.274 and *p*=0.026.

**Table 4:** Lifestyle and dietary habit correlated with urinary OPFRs of children aged 1-17 years (μg/L).

	BDCPP	BCEP	DBEP	DNBP	DPHP	TDCPP	TCEP	TBEP	TNBP	TPHP	ΣOPFRs
Frequency of eating out											
< 7 times/week (n=41)	0.316 ± 0.940	0.539 ± 0.689	0.319 ± 0.570	0.002 ± 0.007	0.424 ± 0.297	0.044 ± 0.093	0.115 ± 0.513	0.473 ± 0.695	0.030 ± 0.035	0.024 ± 0.051	2.287 ± 1.893
≥ 7 times/week (n=21)	0.321 ± 0.730	0.403 ± 0.463	0.146 ± 0.216	0.007 ± 0.013	0.858 ± 1.416	0.022 ± 0.068	0.643 ± 1.083	0.188 ± 0.273	0.045 ± 0.046	0.017 ± 0.035	2.643 ± 1.998
*p*-value ^a^	0.676	0.61	0.304	0.208	0.693	0.438	0.008**	0.042*	0.232	0.756	0.499
Freguency of hand-washing habit before eating											
Usually or always (n=37)	0.360 ± 1.017	0.473 ± 0.708	0.237 ± 0.608	0.002 ± 0.008	0.687 ± 1.093	0.048 ± 0.099	0.356 ± 0.847	0.363 ± 0.491	0.040 ± 0.038	0.026 ± 0.054	2.646 ± 1.916
Absolute (n=25)	0.288 ± 0.609	0.264 ± 0.189	0.219 ± 0.367	0.007 ± 0.013	0.424 ± 0.303	0.020 ± 0.059	0.201 ± 0.697	0.388 ± 0.744	0.022 ± 0.029	0.016 ± 0.031	2.073 ± 1.906
*p*-value ^a^	0.771	0.616	0.203	0.325	0.96	0.604	0.362	0.658	0.049*	0.716	0.13
Frequency of daily consumption of beverages from plastic cups											
less or never, (n=25)	0.182 ± 0.507	0.503 ± 0.732	0.339 ± 0.688	0.001 ± 0.006	0.006 ± 0.011	0.233 ± 0.173	0.027 ± 0.075	0.262 ± 0.752	0.354 ± 0.412	0.035 ± 0.037	0.031 ± 0.058
≥ 1 cup/day (n=35)	0.409 ± 1.048	0.502 ± 0.554	0.219 ± 0.279	0.001 ± 0.006	0.571 ± 0.792	0.053 ± 0.101	0.160 ± 0.584	0.560 ± 1.114	0.198 ± 0.567	0.009 ± 0.014	0.014 ± 0.034
*p*-value ^a^	0.399	0.761	0.832	0.781	0.905	0.279	0.645	0.357	0.861	0.344	0.284
Frequency of daily consumption of microwaved food											
less or never, (n=43)	0.400 ± 0.999	0.488 ± 0.644	0.312 ± 0.563	0.002 ± 0.007	0.520 ± 0.627	0.044 ± 0.091	0.148 ± 0.553	0.504 ± 1.065	0.205 ± 0.535	0.017 ± 0.029	0.013 ± 0.032
≥ 1 time/day (n=16)	0.104 ± 0.285	0.572 ± 0.606	0.164 ± 0.205	0.005 ± 0.011	0.809 ± 1.374	0.024 ± 0.077	0.426 ± 0.958	0.467 ± 0.687	0.036 ± 0.043	0.030 ± 0.054	2.447 ± 2.070
*p*-value ^a^	0.481	0.627	0.707	0.282	0.885	0.467	0.172	0.186	0.591	0.084	0.597
Frequency of weekly intake of seafood											
< 2 times/week (n=22)	0.481 ± 1.208	0.458 ± 0.541	0.191 ± 0.380	0.001 ± 0.006	0.688 ± 1.132	0.038 ± 0.095	0.290 ± 0.773	0.504 ± 0.749	0.035 ± 0.043	0.030 ± 0.063	2.980 ± 2.298
≥ 2 times/week (n=40)	0.249 ± 0.615	0.264 ± 0.189	0.219 ± 0.367	0.007 ± 0.013	0.522 ± 0.697	0.036 ± 0.081	0.296 ± 0.806	0.302 ± 0.497	0.031 ± 0.031	0.018 ± 0.034	2.104 ± 1.620
*p*-value ^a^	0.842	0.939	0.068	0.953	0.453	0.93	0.94	0.104	0.881	0.523	0.133
Frequency of weekly intake of large fish											
less or never, (n=19)	0.374 ± 1.137	0.486 ± 0.738	0.197 ± 0.265	0.002 ± 0.007	0.004 ± 0.010	0.248 ± 0.211	0.027 ± 0.069	0.335 ± 0.825	0.285 ± 0.471	0.031 ± 0.037	0.019 ± 0.035
≥1 time/week (n=38)	0.317 ± 0.758	0.506 ± 0.576	0.193 ± 0.378	0.001 ± 0.006	0.781 ± 1.208	0.042 ± 0.098	0.039 ± 0.097	0.312 ± 0.824	0.643 ± 1.025	0.041 ± 0.054	0.018 ± 0.029
*p*-value ^a^	0.818	0.718	0.602	0.983	0.282	0.523	0.833	0.434	0.836	0.946	0.672
Frequency of daily meat servings											
< 3 times/week (n=55)	0.373 ± 0.916	0.248 ± 0.202	0.204 ± 0.400	0.508 ± 0.622	0.197 ± 0.188	0.331 ± 0.830	0.289 ± 0.501	0.035 ± 0.040	0.023 ± 0.048	2.457 ± 1.907	0.000 ± 0.000
≥ 3 times/week (n=8)	0.511 ± 0.651	0.263 ± 0.503	0.001 ± 0.006	1.046 ± 1.829	0.028 ± 0.072	0.385 ± 0.611	0.297 ± 0.693	0.038 ± 0.033	0.016 ± 0.035	2.004 ± 1.976	0.000 ± 0.000
*p*-value ^a^	0.17	0.636	0.913	0.502	0.591	0.453	0.253	0.465	0.614	0.72	0.322

a Mann–Whitney U test.

**Table 5:** The Spearman’s rho correlation coefficients between urinary OPFRs and neonatal birth outcomes (n=66).

Chemicals	CRE^a^	QI^a^	AS1^a^	AS5^a^	BL^a^	BW^a^	HC^a^	CC^a^	AC^a^	GA^a^
BDCPP	0.179^b^ (0.152)^c^	-0.156 (0.212)	0.036 (0.773)	0.045 (0.722)	-0.249* (0.044)	-0.228 (0.066)	-0.12 (0.304)	-.0261* (0.034)	-0.22 (0.071)	-0.143 (0.252)
BCEP	0.126 (0.312)	-0.02 (0.865)	0.126 (0.315)	-0.166 (0.183)	0.157 (0.208)	0.082 (0.515)	0.116 (0.354)	0.106 (0.399)	0.085 (0.497)	0.002 (0.990)
DBEP	0.100 (0.423)	-0.119 (0.341)	0.023 (0.854)	0.042 (0.740)	-0.252* (0.041)	-0.219 (0.077)	-0.215 (0.083)	-0.154 (0.216)	-0.108 (0.386)	0.051 (0.687)
DNBP	-0.100 (0.422)	0.022 (0.862)	0.005 (0.965)	0.023 (0.854)	0.017 (0.889)	0.016 (0.899)	-0.124 (0.319)	-0.015 (0.904)	-0.009 (0.946)	0.122 (0.328)
DPHP	0.109 (0.386)	-0.091 (0.469)	0.000 (1.000)	0.039 (0.756)	-0.141 (0.257)	-0.152 (0.223)	-0.140 (0.263)	-0.061 (0.629)	-0.041 (0.742)	-0.043 (0.733)
TDCPP	-0.229 (0.065)	0.012 (0.925)	0.051 (0.683)	0.063 (0.614)	-0.089 (0.479)	-0.053 (0.672)	-0.227 (0.067)	-0.045 (0.719)	0.006 (0.963)	0.013 (0.918)
TCEP	-0.030 (0.809)	0.234 (0.058)	0.038 (0.764)	-0.037 (0.769)	0.231 (0.062)	0.274* (0.026)	0.157 (0.209)	0.185 (0.137)	0.068 (0.586)	0.235 (0.057)
TBEP	-0.077 (0.538)	-0.156 (0.211)	-0.012 (0.926)	0.024 (0.850)	-0.182 (0.143)	-0.199 (0.109)	-0.143 (0.252)	-0.168 (0.178)	-0.124 (0.32)	0.010 (0.936)
TNBP	0.046 (0.711)	-0.024 (0.850)	-0.007 (0.954)	0.046 (0.714)	0.05 (0.692)	0.001 (0.993)	-0.055 (0.662)	0.097 (0.438)	0.047 (0.708)	0.025 (0.84)
TPHP	-0.120 (0.337)	-0.009 (0.944)	0.015 (0.907)	0.035 (0.779)	0.074 (0.554)	0.033 (0.794)	-0.056 (0.656)	-0.055 (0.659)	0.001 (0.993)	0.126 (0.312)
ΣOPFRs	0.075 (0.548)	-0.023 (0.858)	0.076 (0.543)	-0.034 (0.788)	-0.047 (0.711)	-0.04 (0.729)	-0.077 (0.537)	-0.03 (0.809)	-0.054 (0.669)	0.145 (0.246)

a CRE: creatinine (9.84 ± 4.19 mg/dL); Quetelet’s index (QI) (12.0 ± 1.17 kg/m^2^); Apgars score (AS1) 1min (8.97± 0.173); Apgars score (AS5) 5 min (9.95±0.210); Birth length (BL) (49.3 ± 2.45 cm); Birth weight (BW) (2.94 ± 0.464 kg); Head circumference (HC) (49.3 ± 2.45 cm); Chest circumference (CC) (31.8 ± 2.04 cm); Abdominal circumference (AC) (29.4 ± 2.18 cm); Gestational age (GA) (37.9± 1.34 weeks)

b Spearman’s rho correlation coefficient.

c (p-value).

^*^p< 0.05, ^**^p< 0.01, ^***^ p< 0.001.

EDIs of individual OPFRs, such as TDCPP, TCEP, TBEP, TNBP, and TPHP, were as follows: (1) newborns: 19.7 ± 97.0, 1170 ± 1660, 493 ± 160, and 25.4 ± 100, and 518 ± 777 ng/kg bw/day for TDCPP, TCEP, TBEP, TNBP, and TPHP, respectively; (2) young children aged 1-5 yrs: 22.5 ± 48.3, 246 ± 302, 12.4 ± 19.9, and 0.219 ± 0.955, and 180 ± 249 ng/kg bw/day for TDCPP, TCEP, TBEP, TNBP, and TPHP, respectively; (3) school children aged 6-10 yrs: 6.74 ± 18.7, 53.8 ± 69.7, 2.44 ± 4.26, and 0.234 ± 0.622, and 66.8 ± 64.3 ng/kg bw/day for TDCPP, TCEP, TBEP, TNBP, and TPHP, respectively; (4) adolescents aged 11-17 yrs: 2.09 ± 5.36, 94.9 ± 101, 2.00 ± 2.87, and <LOD, and 84.7 ± 174 ng/kg bw/day for TDCPP, TCEP, TBEP, TNBP, and TPHP, respectively.

**Table 6** shows concentrations of OPFRs in the urine of individuals under 18yrs in the general population compared with previous measurements in other studies from several locations. Previous studies have shown that BDCPP and DPHP are the most dominant OPFRs (20–27, 29, 31–34, 36, 38). The concentrations of BDCIPP (0.279 μg/L) and DPHP (0.628 μg/L) measured in our study for the 1-5 yrs age group were found to be lower than those reported in previous studies from the United States and Australia (22, 23, 33). Similarly, the concentration of BDCPP (0.053 μg/L) in the newborn group measured in our study was lower than that reported by Hoffman et al. in 2015 and 2017 (29, 36). In 2018, Ospina et al. (31) found children aged 6 to 11 yrs concentrations of BDCPP and DPHP, of 2.25 μg/L and 1.69 μg/L, respectively, which was higher than the concentrations found in our study. The lower concentrations found in our study than those previously reported for newborns and young children suggest that exposure to BDCPP and DPHP in this age group may have decreased over time.

**Table 6:** Global data for urinary OPFRs and OPFR metabolites in the general population under 18 years old.

Region (country)	Reference	Age (years)	OPFRs											
			BCEP	BCIPP	BDCIPP	BBOEP	DNBP	DPHP	DBEP	TDCPP	TCEP	TBEP	TNBP	TPHP
Germany	Fromme et al. (27)	1.8-6.7	NA	NA	NA	NA	0.20	0.80	2.0	NA	NA	NA	NA	NA
Norway	Cequier et al. (24)	NA	NA	NA	(0.20)	NA	NA	(0.57)	NA	NA	NA	NA	NA	NA
U.S.	Butt et al. (22)	1-5	NA	NA	(5.60)	NA	NA	(3.0)	NA	NA	NA	NA	NA	NA
Norway	Cequier et al. (25)	NA	NA	NA	0.23	NA	NA	1.10	NA	NA	NA	NA	NA	NA
Australia	Van den Eede et al. (33)	0-5	NA	NA	(1.00)	(< 0.35)	(< 0.43)	(24.4)	NA	NA	0.35	NA	NA	NA
U.S.	Hoffman et al. (29)	0-1.5	NA	NA	(2.30)	NA	NA	(1.0)	NA	NA	NA	NA	NA	NA
U.S.	Butt et al. (23)	0-5.8	NA	NA	7.70	NA	NA	1.90	NA	NA	NA	NA	NA	NA
U.S.	Hoffman et al. (36)	0-1.5	NA	NA	(2.29)^c^	NA	NA	NA	NA	NA	NA	NA	NA	NA
U.S.	Thomas et al. (32)	1.3-1.5	NA	NA	(6.81)	NA	NA	NA	NA	NA	NA	NA	NA	NA
U.S.	Ospina et al. (31)	6.0-11	(0.66)	(0.27)	(2.25)	NA	(0.27)	(1.69)	NA	NA	NA	NA	NA	NA
South China	Chen et al. (26)	Jun-14	(3.25)	(0.21)	(0.12)	(0.06)	(0.16)	(0.40)	NA	NA	NA	NA	NA	NA
China	Zhang et al. (34)	0-5	0.85 ^a^	0.69	0.08	0.04	0.06	0.27	NA^b^	NA	NA	NA	NA	NA
Australia	He et al. (28)	0-2.4	< 0.017	1.5	7.8	0.22	NA	2.3	NA	0.056	<0.054	1.1	NA	NA
Japan	Bamai et al. (20)	7	NA	(0.007)	(0.3)	(0.25)	(0.071)	(0.67)	NA	NA	(0.05)	NA	NA	NA
Japan	Bastiaensen et al. (21)	7-12	NA	(0.28)	(0.12)	(0.34)	(0.05)	(0.51)	NA	NA	NA	NA	NA	NA
Taiwan	The present study	Newborn	(0.705)	NA	(0.053)	NA	(0.04)	(0.395)	(0.406)	(0.009)	1.13	0.424	0.043	0.047
Taiwan	The present study	1~5	(0.680)	NA	(0.279)	NA	90.00158)	(0.628)	(0.469)	(0.0421)	0.273	0.573	0.0359	0.0189
Taiwan	The present study	6~10	(0.293)	NA	(0.165)	NA	(0.00333)	(0.461)	(0.182)	(0.0193)	0.303	0.221	0.0276	0.011
Taiwan	The present study	11~18	(0.517)	NA	(0.514)	NA	< LOD	(0.585)	(0.149)	(0.045)	0.203	0.251	0.0368	0.0355

a Median.

b Not available or not analysed.

c Numbers in parentheses are the means.

## 4 Discussion

This is the first study to report urinary OPFRs and OPFR metabolites in the Taiwanese population under 18 years old. The findings suggest that exposure to OPFRs may be a concern across a wide range of age groups. **Table 1** and **Figure 1** show that the distribution of OPFRs and OPFR metabolites in newborns’ urine is different from those in the other age groups. For example, BDCPP composed 1.63% and 9.12-22.2% of Σ_10_ OPFRs in newborns’ and the other age groups’ urine, respectively, while TCEP comprised 34.8% and 8.67-16.9% of Σ_10_ OPFRs in newborns’ and the other age groups’ urine, respectively. This indicates that OPFRs and their metabolites distributed in newborns’ urine might differ from those in urine from the other age categories. These organophosphate compounds are commonly used as flame retardants in consumer products and the findings suggest that newborns may be especially vulnerable to exposure to these chemicals, particularly for breastfed infants via breast milk. Tsai et al. (2022) (35) indicated that urinary Σ_10_ OPFRs in patients with chronic kidney disease (CKD) (n=166 at the age of 69) in southern Taiwan were detected as the median of 2.07 μg/g creatinine (Cr), including the predominant urinary OPFRs of TBEP, BCEP, Bis(2-butoxyethyl) phosphate (BBOEP), and DPHP. Urinary OPFRs in a broad-spectrum young population in the present study differ from that of the Taiwanese patients with CKD from Tsai’s study (35). Human exposure to OPFRs is mainly from dietary and indoor dust, particularly for newborns and young children, who spend longer periods of time in the home. According to the current data by Chang, for southern Taiwan (30), TCPP contributes 39.0% to dust ΣOPFRs, followed by Triethyl phosphate (TEP) and Tri (isobutyl) phosphate (TiBP). Our results are not comparable to those of Lu (30) due to differences in the detection methods of OPFR species. Lu also assessed the hazard of residential indoor air and house dust in Taiwan, with average daily exposure doses of 152, 137, and 142 ng/kg/day for children aged 3-5 yrs, boys aged 6-12 yrs, and girls aged 6-12 yrs, respectively. Our findings, indicating that Σ_10_ OPFRs in urine from children aged 3-5 yrs are higher than those from children aged 6-12 yrs, are consistent with the estimated daily doses of non-dietary OPFRs reported by Lu (30). Urinary levels of DPHP and BDCPP were comparable, and urinary TCEP was higher in the present study than in the Hokkaido cohort of Japanese school children aged 7 yrs (20). DPHP is frequently detected and predominates in urine from our population, with a level comparable to previous studies from Japan (20, 21), China (26), and Europe (25, 27). Dodson et al. (2014) indicated that DPHP was not a specific biotransformation product of TPHP or the metabolites of 2-ethylhexyldiphenyl phosphate (EHDPHP) and resorcinol bis-diphenyl phosphate (RDP) (39). The Hokkaido cohort study examined associations between OPFR metabolites and urinary oxidative stress biomarkers (8-hydroxy-2′-deoxyguanosine, hexanoyl-lysine (HEL), and 4-hydroxynonenal), finding that urinary DPHP was significantly and positively correlated with HEL, which is recognized as lipid hydroperoxide-modified biomarkers oxidized from fatty acids (20).

There were no significant differences in urinary OPFRs and OPFR metabolites among the four age groups (**Table 1**). Most previous studies also found non-significant differences in OPFR metabolites between the age groups. Urinary levels of DPHP (6-11 yrs vs 12-18 yrs: 1.69 vs 1.41 μg/L), BCEP (0.662 vs 0.602 μg/L), and DNBP (0.272 vs 0.207 μg/L) in school children in the 6-11 yrs age group were not significantly higher than those in the 12-18 yrs adolescent group, but urinary BDCPP (2.25 vs 1.34 μg/L, *p* < 0.001) was statistically significant (31). According to several mother-children paired studies, urinary OPFR metabolites in children showed several folds higher than those in mothers (22, 23, 25). Based on our findings, the distribution of urinary OPFRs and OPFR metabolites in newborns significantly differed from those whose urine was from young children, school children, and adolescents (**Figures 1A, B**). Urinary OPFRs and their metabolites did not present significant differences between male and female subjects in a broad-spectrum young population, as shown in **Figure 1C**. Urinary Σ_10_ OPFRs in male adults were not significantly different from that in female adults from Taiwanese CKD patients (35). In a German study, urinary OPFR metabolites did not show a significant difference between boys and girls at the age of 22-80 months (n = 312) (27). For American infants, urinary BDCPP and DPHP did not show significant differences by sex (29), while data from the NHANES also showed non-significant differences by sex in urinary BDCPP, DPHP, BCEP, and DNBP (31). **Table 2** presents the correlations between OPFRs and OPFR metabolites in the urine of newborns. There were high correlation coefficients (r > 0.5) in the pairs of TBEP and its metabolite of DBEP (r = 0.816, *p* < 0.001), as well as TNBP and its metabolite of DNBP (r = 0.509, *p* < 0.001). This is probably due to different metabolizing efficiencies and rates of OPFRs in newborns, indicating that TNBP and TBEP as well as their metabolites of DNBP and DBEP may have longer half-lives than the other OPFRs and OPFR metabolites. The OPFR of TPHP was significantly correlated with other OPFRs, such as TDCCP (r = 0.458, *p* < 0.001), TCEP (r = -0.279, *p* = 0.023), TBEP (r = 0.325, *p* = 0.008), and TNBP (r = 0.244, *p* = 0.048). If only OPFRs are considered in the urine of newborns, urinary OPFRs are probably predicted by TPHP. **Table 3** shows the high correlation coefficient (r > 0.5) of TBEP and DBEP (r = 0.845, *p* < 0.001) in urine collected from a broad-spectrum young population, except for newborns. **Figure 2B** does not show the significant correlations between demographic parameters and urinary OPFRs in the general population of young individuals at the age 1-17 yrs (n=69). In **Table 4**, lifestyles and dietary habits of children are grained from the questionnaire. Among the variables listed in **Table 4**, frequency of eating out and frequency of hand-washing before eating are presented as significant in certain OPFRs such as TCEP, TBEP and TNBP. To our amazement, TCEP, TBEP, and TNBP were parent OPFRs and their metabolites (BCEP, DBEP, and DNBP) did not perform any associations with the questionnaires’ variables in the present study. Based on our results, for the newborn group (n=66), certain OPFR metabolites, such as BDCPP, DBEP, and TDCPP (the parent compound of BDCPP), show the negatively significant (*p*<0.05) or borderline-significant (*p*<0.1) relationship with certain birth outcomes such as birth length, birth weight, head circumference, chest circumference, or abdominal circumference. Urinary BDCPP in newborns was negatively correlated with birth length (r=-0.249, *p*=0.044), birth weight (r=-0.228, *p*=0.066), chest circumference (r=-0.261, *p*=0.034), and abdominal circumference (r=-0.223, *p*=0.071). Urinary DBEP had negative associations with birth length (r=-0.252, *p*=0.041), birth weight (r=-0.219, *p*=0.077), and head circumference (r=-0.-215, *p*=0.083). TDCPP levels in the urine of newborns were only shown to have a borderline-significant relationship with urinary creatinine (r=-0.229, *p*=0.065) and head circumference (r=-0.227, *p*=0.067). Inversely, urinary TCEP (the parent compound of BCEP) was positively linked to Quetelet’s index (r=0.234, *p*=0.058), birth length (r=0.231, *p*=0.062), birth weight (r=0.274, *p*=0.026), and gestational age (r=0.235, *p*=0.057). Based on our findings, the abundant compounds of BCEP and DPHP in the urine of newborns present non-significant or non-borderline-significant correlations with the variables of birth outcomes.

EDIs of Σ_5_OPFRs (sum of TDCPP, TCEP, TBEP, TNBP, and TPHP) were 2230, 461, 130, and 184 ng/kg bw/day for newborns, children aged 1-5 yrs, children aged 6-10 yrs, and adolescents aged 11-17 yrs, respectively. The Σ_5_OPFRs EDI for newborns was 4.83-17.2 times higher than those for the other age groups. Van den Eede et al. (2011) (40) found that the reference doses (RfDs) of TDCIPP, TNBP, TCEP, and TPHP were 1500, 2400, 2200, and 7000 ng/kg bw/day, respectively, for a range of cohorts grouped by age and sex. All EDIs of TDCIPP, TNBP, TCEP, and TPHP for the young population in the present study were below the RfDs. This indicates that the high body burden of OPFRs for newborns in the present study is still lower than critical values. Lu et al. (2023) (30) assessed the Σ_11_OPFRs EDI via house dust and indoor air in residential houses to be 152 ng/kg bw/day for Taiwanese children aged 3-5 yrs and 137-142 ng/kg bw/day for Taiwanese children aged 6-12 yrs. Young and school-age children’s exposure to OPFRs is primarily through non-dietary (i.e., hand-mouth behavior) and dietary pathways. Our EDI for urinary metabolite excretion had a magnitude comparable to the values from Lu’s study, which were assessed by the routes of indoor matrices (30). Our EDI had the same order of magnitude as those in Chinese children (8-12 yrs and 6-14 yrs, of 474 and 172 ng/kg bw/day, respectively, n=411) (26), but lower than those in German children (TNBP: 30 ng/kg bw/day of the median, n=312) (27), and American infants at 2-18 months (110-330 ng/kg bw/day of geometric means, n=43) (36) as estimated from excreted OPFR metabolites. The EDIs of TCEP, TDCPP, and TPHP for newborns in the present study were slightly higher than those for infants in the USA (n=65) (34), considering only the assessment from urine excretion. Apart from indoor dust or indoor air, food is a major route for the young population’s exposure to OPFRs. Breastfed infants are a unique population to estimate OPFRs due to their primary consumption of breast milk. Since there is currently no data on OPFRs and their metabolites in Taiwanese breast milk, levels of OPFRs and OPFR metabolites in breast milk were referenced from previous studies. Chen et al. (2021) (41) found a mean Σ_13_OPFRs value of 21.7 ng/mL (587 ng/g lipid) (median=10.6 ng/mL or 157 ng/g lipid) for breastmilk OPFRs in Beijing mothers (n=105), while the predominant congeners of TEHP, TPHP, and EHDPP had medians of 1.47, 1.07, and 0.844 ng/mL, respectively. OPFRs in breast milk were also measured in US mothers, with 3.6, 1.44, 0.569, and 0.539 ng/mL for Σ_14_OPFRs, TBOEP, TIBP, and TNBP, respectively (42). Based on the reports by Chen (41) and Ma (42), OPFRs and their metabolites have high magnitudes in breast milk.

As seen in **Figure 1A** and **Table 1**, the newborns had higher Σ_10_OPFRs than the toddlers, young children, school children, and adolescents, and the profiles of urinary OPFRs in newborns were differently distributed from those in the other age groups. Newborns’ exposure to OPFRs was probably mainly from the maternal breast milk in the studies by Blum (1), Chen (41), and Ma (42) as well as in the present study. Humans, including the young population, spend more than 90% of their time indoors. For the other age groups, such as toddlers, young children, school children, and adolescents, the profiles of urinary OPFRs were similar, indicating that these age groups possibly have the same exposure sources. Recently, two published articles investigated OPFRs in the indoor environment of Taiwanese residents, including air, PM_2.5_, and house dust (30, 43). Liu et al. (2023) assessed children’s daily average exposure to OPFRs and found that toddlers aged 3-5 yrs had a daily average exposure of 152 ng/kg/day, boys aged 6-12 yrs had 137 ng/kg/day, and girls aged 6-12 yrs had a daily average exposure of 142 ng/kg/day, from air inhalation and dust ingestion (43). This report also indicated airborne TEP, TCPP, and TBOEP mainly from wooden materials, house dust of TBP, TDCPP, and TBOEP probably from plastic materials, TEP and TEHP in house dust possibly from foam and textile materials as well as textile and plastic materials, respectively, in the indoor environment (43). Liu et al. (2023) (30) revealed that the sources of OPFRs indoors and outdoors primarily originated from the indoor environment.

To the best of our knowledge, this research is the first study to announce urinary OPFRs and OPFR metabolites in a broad-spectrum young population. It also presents the first findings for urinary OPFRs in newborns. **Table 6** shows that global data for the urine of children indicate the diverse distribution and variation of OPFR magnitudes. The different commercial formulations of OPFRs used in the different regions and different dietary habits in the different populations may have led to the geographic and population differences for OPFRs.

## 5 Conclusions

The distribution of OPFRs and their metabolites in the urine of newborns differs from that in urine samples from other age categories. To our knowledge, this is the first article investigating urinary OPFR metabolite levels in a broad-spectrum young population. Though there are higher exposure rates for both newborns and preschool children, little is known about their exposure levels or factors leading to exposure in the young population. Based on the present study and previous research, breastfeeding is the major pathway for OPFRs exposure for newborns while in other age groups (toddlers, young children, school children, and adolescents), the children’s exposure to OPFRs is mainly from consumer products via air inhalation and house dust ingestion. Urinary OPFRs are not significantly correlated with creatinine in the urine of newborns. Further study should clarify the exposure levels and factor relationships. There is a pressing need to improve our understanding of the potential health impacts of OPFRs, given their widespread use and persistent presence in the environment.

## Data availability statement

The raw data supporting the conclusions of this article will be made available by the authors, without undue reservation.

## Ethics statement

The studies involving human participants were reviewed and approved by Chang Gung Memorial Hospital (CGMH) (202001028A3). Written informed consent to participate in this study was provided by the participants’ legal guardian/next of kin.

## Author contributions

F-SC and C-CC are co-first authors and contributed equally to this manuscript. They participated in the study design, patient recruitment, reviewing references, and drafting the manuscript. C-CT, J-HL, H-LY, C-MC, W-TH, K-FT, F-JC, C-TK, S-HL, C-CW, Y-CO, W-CL, Y-TC, and FH participated in patient recruitment or laboratory analyses. H-RC and L-JW are co-corresponding authors and contributed equally to this manuscript. They participated in study design, laboratory analyses, as well as writing the manuscript draft and revisions. All authors contributed to the article and approved the submitted version.
